# Genome Sequence of a Cynomolgus Macaque Adenovirus (CynAdV-1) Isolate from a Primate Colony in the United Kingdom

**DOI:** 10.1128/genomeA.01193-16

**Published:** 2016-11-03

**Authors:** Zhiwei Zeng, Jing Zhang, Shuping Jing, Zetao Cheng, Silvia Bofill-Mas, Carlos Maluquer de Motes, Ayalkibet Hundesa, Rosina Girones, Donald Seto, Qiwei Zhang

**Affiliations:** a Biosafety Level-3 Laboratory, School of Public Health, Southern Medical University (Guangdong Provincial Key Laboratory of Tropical Disease Research), Guangzhou, Guangdong, China; b Section of Microbiology, Virology and Biotechnology, Department of Genetics, Microbiology and Statistics, University of Barcelona, Barcelona, Spain; c Bioinformatics and Computational Biology Program, School of Systems Biology, George Mason University, Manassas, Virginia, USA; d Department of Ophthalmology, Howe Laboratory, Massachusetts Eye and Ear Infirmary, Harvard Medical School, Boston, Massachusetts, USA

## Abstract

The genome sequence of a simian adenovirus from a cynomolgus macaque, denoted CynAdV-1, is presented here. Phylogenetic analysis supports CynAdV-1 in an independent clade, comprising a new simian adenovirus (SAdV) species. These genome data are critical for understanding the evolution and relationships of primate adenoviruses, including zoonosis and emergent human pathogens.

## GENOME ANNOUNCEMENT

Adenoviruses infect a broad range of vertebrate hosts, including humans and nonhuman primates (1). To date, at least 69 different human adenoviruses (HAdVs) and 25 simian adenoviruses (SAdVs) have been described and grouped into eight human (A to G) and one simian (A) species (1, 2). Additionally, adenoviruses are reported in cross-species zoonotic transmissions, for example, between nonhuman primates and human hosts (3–7). In one seminal example, HAdV-4, a major human respiratory pathogen and one of the original identified and characterized human respiratory adenoviral pathogens, has been characterized as a chimpanzee virus that has transferred hosts stably through zoonosis (6–9). As human pathogens, HAdVs can produce latent and asymptomatic infections as well as cause mild to severe disease, including death. They can affect the respiratory, ocular, gastrointestinal, metabolic, and urogenital systems (10–12). Over the last decade, HAdVs, and especially SAdVs, have become important tools for improving human health as gene delivery vectors for gene therapy and for vaccine development (13, 14). Given the possibility of preexisting immunity against HAdVs in humans, SAdVs are a valuable alternative, as they are presumably not found in humans (15–17).

SAdVs have been isolated from apes, including chimpanzees (18, 19), and monkeys, including macaques and baboons (4, 20). Cross-species zoonotic transmissions have been reported, with both hosts retaining antigenic profiles of the event (3, 4). These zoonotic events are important, as genome recombination is documented among HAdVs as a pathway of generating novel human pathogens that cause acute respiratory and epidemic keratoconjunctivitis diseases (21–23); therefore, genome recombination with SAdVs may be another pathway for the genesis of emergent human pathogens (5). In this context, the further and additional characterization of SAdVs from apes and monkeys is of critical importance for understanding SAdVs as human pathogens and as vectors in the development of gene therapy and vaccine vectors.

Cynomolgus adenovirus 1/UK/UK-1/2004 was isolated from a colony of cynomolgus macaques (*Macaca fascicularis*) (20). Its genome (35,555 nucleotides) was sequenced using an Ion Torrent on the Personal Genome Machine and assembled with CLC Genomics Workbench 7 (CLC bio; Aarhus, Denmark). It yielded 12.8 Mb filtered reads with an average read length of 180 bp, 110× coverage, and an *N*_50_ of 1,226 bp. This was supplemented with the Sanger chemistry method to fill gaps and ambiguous sequences. Initial gene analyses by Maluquer de Motes et al. (20) and preliminary whole-genome analysis indicate that CynAdV-1 is in a unique subclade, branching with SAdV-6 and -3, and that all three are contained in a larger clade that includes HAdV species A, F, and G. Discussions of the concept of HAdV and SAdV “species” are ongoing; this CynAdV-1 genome sequence will add to the discussions and understanding of the relationships between HAdVs and SAdVs, including a combined and integrated tree with proposed “primate AdV” species, as the HAdVs and SAdVs are phylogenetically close and all species branch from one common phylogenetic tree.

### Accession number(s).

Genome data for cynomolgus adenovirus 1/UK/UK-1/2004 are available in the GenBank database under the accession number KT013209.
